# Reduction of the off-pathway iron-sulphur cluster N1a of *Escherichia coli* respiratory complex I restrains NAD^+^ dissociation

**DOI:** 10.1038/s41598-017-09345-4

**Published:** 2017-08-18

**Authors:** Emmanuel Gnandt, Johannes Schimpf, Caroline Harter, Jo Hoeser, Thorsten Friedrich

**Affiliations:** Albert-Ludwigs-Universität, Institut für Biochemie, Albertstr. 21, Chemie-Hochhaus, 79104 Freiburg i. Br., Germany

## Abstract

Respiratory complex I couples the electron transfer from NADH to ubiquinone with the translocation of protons across the membrane. The reaction starts with NADH oxidation by a flavin cofactor followed by transferring the electrons through a chain of seven iron-sulphur clusters to quinone. An eighth cluster called N1a is located proximally to flavin, but on the opposite side of the chain of clusters. N1a is strictly conserved although not involved in the direct electron transfer to quinone. Here, we show that the NADH:ferricyanide oxidoreductase activity of *E. coli* complex I is strongly diminished when the reaction is initiated by an addition of ferricyanide instead of NADH. This effect is significantly less pronounced in a variant containing N1a with a 100 mV more negative redox potential. Detailed kinetic analysis revealed that the reduced activity is due to a lower dissociation constant of bound NAD^+^. Thus, reduction of N1a induces local structural rearrangements of the protein that stabilise binding of NAD^+^. The variant features a considerably enhanced production of reactive oxygen species indicating that bound NAD^+^ represses this process.

## Introduction

NADH:ubiquinone oxidoreductase, respiratory complex I, couples electron transfer from NADH to ubiquinone with the translocation of protons across the membrane^1–4^. The complex shows a two-part structure with a peripheral arm containing the redox groups and a membrane arm catalysing proton translocation. Recently, the structures of the bacterial and mitochondrial complexes were solved at molecular resolutions revealing the putative electron pathway in the peripheral arm and four presumptive proton channels in the membrane arm^5–7^. The coupling of electron transfer and proton translocation remains largely elusive; however, quinone reduction seems to play a central role^8^.

The initial step of enzyme catalysis is NADH oxidation by a non-covalently bound flavin mononucleotide (FMN) through hydride transfer^9, 10^. The reduced flavin is re-oxidised by a chain of seven iron-sulphur (Fe/S) clusters transferring the electrons to the substrate quinone^11^. An eighth Fe/S cluster, N1a, is located in electron transfer distance to FMN on the opposite site of the Fe/S cluster chain. In *Escherichia coli*, the first electron from the FMNH_2_/FMNH^•^ couple is transferred down the Fe/S cluster chain to the quinone, while N1a participates in electron transfer by accepting one electron from the FMNH^•^/FMN couple. Upon re-oxidation of the Fe/S clusters of the chain, the electron stored on N1a is fed into the chain to complete the two-electron reduction of the quinone^11^. Surprisingly, despite considerable efforts, N1a of the mitochondrial complex I appears to be resistant to reduction^12^.

Re-oxidation of the reduced flavin is achieved *in vitro* by artificial hydrophilic electron acceptors such as potassium hexacyanoferrate(III) (ferricyanide or FeCN)^13^. The reaction follows a ping-pong-pong mechanism with double substrate inhibition. Here, flavin is reduced by NADH and re-oxidised after NAD^+^ dissociation by subsequent binding and single-electron reduction of two ferricyanide molecules at the same site^14^. The NADH:ferricyanide oxidoreductase activity is not linked to energy conservation^15^, but it is commonly used to detect the enzyme in fractions obtained during protein purification. In addition, the re-oxidation of reduced flavin by ferricyanide is much faster than by ubiquinone, thus allowing for a detailed investigation of NADH-dependent steps in the mechanism of complex I^10, 13–20^.

Typically, the reaction is started either by adding NADH or the enzyme to the reaction mix. However, it was reported that the NADH:ferricyanide oxidoreductase activity of *E. coli* complex I is strongly diminished when ferricyanide is added last. In other words, initial reduction of the enzyme prior to exposure to the oxidant results in a substantial loss of activity^21, 22^. It was proposed that this effect is due to a conformational change in the active site^21^ or to dissociation of the flavin cofactor^22^. Remarkably, this effect was not observed with complex I from *Thermus thermophilus* ^22^, although the complexes from both bacterial species share a high degree of similarity except for the redox potential of N1a between −250 and −290 mV in *E. coli* ^12, 23^ and −420 mV in *T. thermophilus* ^24^. In this study, we present a refined analysis of the reaction that disclosed a previously unnoticed increase of the initially low activity over time, which is not compatible with the proposed loss of FMN. Instead, the reduction of N1a appears to induce a local structural rearrangement at the NADH binding site supporting an enhanced interaction with the reaction product, NAD^+^. This in turn, is accompanied by a diminished production of reactive oxygen species (ROS) by complex I.

## Results

### NADH:ferricyanide oxidoreductase activity of complex I in membranes

**Figure 1:**
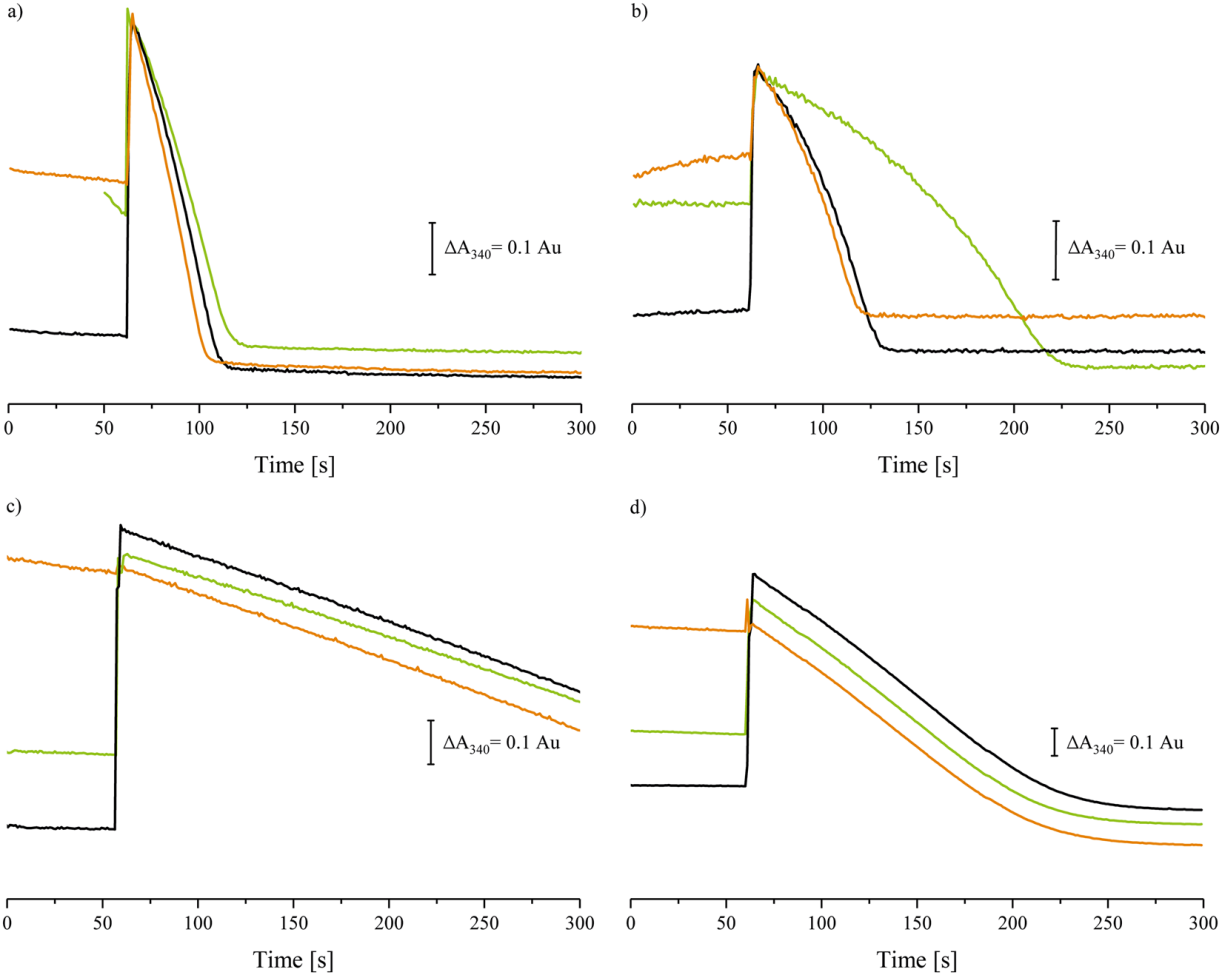
(d-)NADH:ferricyanide oxidoreductase activity of complex I in cytoplasmic membranes from *E. coli* (**a**,**b**) and *T. thermophilus* (**c**) and in bovine heart mitochondrial membranes (**d**). The reaction was either started by an addition of the enzyme (orange), NADH (black) or ferricyanide (green). The assay contained 40 µg membrane proteins in 50 mm MES/NaOH, 50 mm NaCl, pH 6.0 (**a**,**b**), 50 mm Bis-Tris, pH 7.3 (**c**) and 20 mm Tris/HCl, pH 7.5 (**d**). The reaction rates were also determined in the presence of 10 µm piericidin A (**b**). Virtually identical curves were obtained when the membranes were incubated with 20 mm KCN (data not shown). An addition of piericidin A and KCN did not alter the curves shown in (**c**,**d**) and are, therefore, not shown. An offset between the individual traces was introduced for a clearer representation of the curves. The membranes incubated with NADH show significant NADH oxidase activity (**a**, green trace). Due to this, membranes were incubated with NADH for only 10 s before ferricyanide was added.

The NADH:ferricyanide oxidoreductase activity of complex I was first determined with the enzyme in its native membrane (Fig. 1). Ferricyanide was added to *E. coli* cytoplasmic membranes and the reaction was started by an addition of NADH (Fig. 1a). After a short activation phase of about 30 sec, a rapid turnover was observed that remained constant until nearly all substrate was consumed. A similar curve was obtained when the assay contained both substrates and the reaction was started by adding cytoplasmic membranes (Fig. 1a). When cytoplasmic membranes were incubated with NADH first, the NADH concentration decreased due to the intrinsic NADH oxidase activity. Consequently, membranes were only shortly incubated with NADH and the reaction was started by supplying ferricyanide. After a short activation phase the maximum rate was rapidly reached and maintained until substrate depletion (Fig. 1a).

In order to prevent a re-oxidation of complex I by the respiratory chain, we applied inhibitors in the next experiment. Here, the membranes were pre-treated with either piericidin A or KCN, before the reaction was initiated by ferricyanide addition. Piericidin A is a specific inhibitor of complex I addressing the quinone reduction site, while KCN inhibits the *E. coli* terminal oxidases. Hence, both inhibitors block the electron transfer chain and impede NADH oxidase activity. Consequently, we observed a slightly slower rate of NADH consumption indicative of a diminished NADH oxidase activity (Fig. 1a and b). When the reaction was initiated by NADH and enzyme, combined with a prolonged activation period, a fast reaction was observed. This is in contrast to the notably diminished reaction rate we monitored, when the reaction was started by ferricyanide addition. Here, the reaction rate was initially limited to only 20% of the reaction initiated by NADH addition. Intriguingly however, the rate accelerated over time peaking at approximately 55% of the reaction rate initiated by NADH addition before the substrates were depleted (Fig. 1b). In summary, initially reduced complex I cofactor(s) significantly slow down the NADH:ferricyanide oxidoreductase activity but in the course of the reaction these cofactor(s) become partially oxidised^25^, and hence result in a turnover-dependent acceleration of the reaction. Affirmatively, identical curves were obtained when using deamino (d)-NADH, a substrate specific for complex I, confirming that the observed NADH:ferricyanide oxidoreductase activity can be assigned to complex I catalysis.

The same set of experiments was performed with *T. thermophilus* membranes, since the rate of the d-NADH:ferricyanide activity of complex I from this organism was reported to be independent of the order of substrate addition^22^. Here, only d-NADH was used as substrate in order to avoid catalysis by the *T. thermophilus* alternative, non-energy-converting NADH dehydrogenase. Indeed, *T. thermophilus* membranes exhibited similar fast reaction rates, regardless of whether the reaction was initiated by addition of membranes, d-NADH or ferricyanide (Fig. 1c). Repeatedly, the rate of the reaction initiated by ferricyanide reached 90% of that started with the enzyme, as observed with *E. coli* membranes in the absence of inhibitors (Fig. 1a and c). Notably, neither piericidin A nor KCN were found to affect the reaction rates. The differing reaction rates of the *T. thermophilus* and the *E. coli* complex I may arise from the differing redox potential of Fe/S cluster N1a that is approximately 130 to 170 mV lower in *T. thermophilus* than in *E. coli*. In order to validate this hypothesis, another complex with a ‘low potential’ N1a was used, that is found in membranes from bovine heart mitochondria. Again, the rate of the NADH:ferricyanide oxidoreductase reaction was independent from the order of addition of the assay components, both in the absence and in the presence of piericidin A and KCN (Fig. 1d). Thus, the data support the proposal that the initially low activity of the *E. coli* complex I upon reduction by NADH and its subsequent enhancement in a turnover dependent manner by ferricyanide mediated oxidation is indeed related to the high redox potential of its cluster N1a. For a detailed analysis we next monitored the reaction kinetics of isolated *E. coli* complex I.

### NADH:ferricyanide oxidoreductase activity of isolated *E. coli* complex I

**Figure 2:**
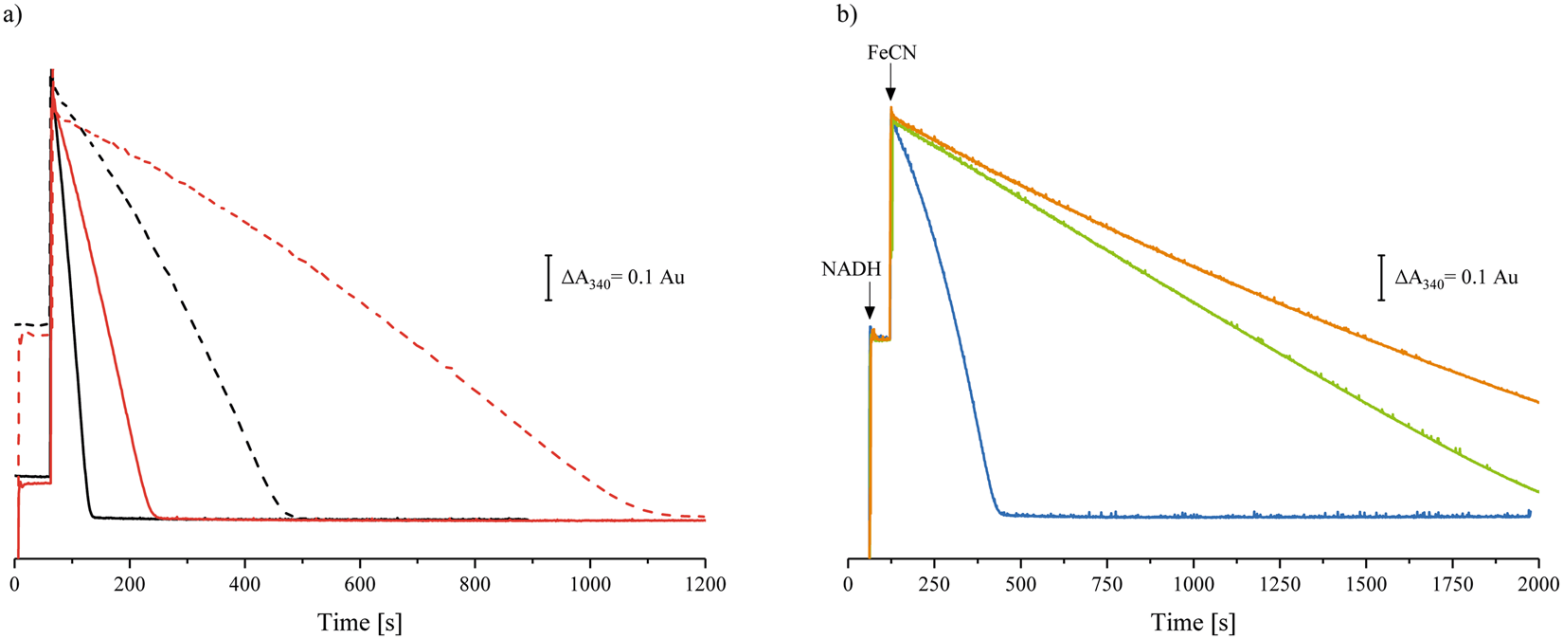
NADH:ferricyanide oxidoreductase activity of isolated *E. coli* complex I. (**a**) Shows the activity of 10 nm (black) and 2 nm (red) isolated complex I. The assays contained 150 µm NADH and 1 mm ferricyanide in 50 mm MES/NaOH, 50 mm NaCl, pH 6.0. The reaction was either started by an addition of NADH (solid line) or ferricyanide (dashed line). (**b**) Shows the activity of 5 nm (blue), 0.9 nm (green) and 0.5 nm (orange) complex I at the experimental conditions described above when the reaction was started by ferricyanide addition (FeCN).

A recent study reported that the initially low activity of the NADH-reduced complex is due to a loss of FMNH_2_ that, once re-oxidised to FMN under oxic conditions, should bind to the apo-complex^22^. The *K*_D_ of FMN to the reduced apo-enzyme was determined to approximately 54 nm ^22^. The NADH:ferricyanide oxidoreductase activity of isolated *E. coli* complex I in the presence of 150 µm NADH and 1 mm ferricyanide as obtained in routine assays is shown in Fig. 2. The protein exhibited an activity of 60–70 µmol NADH/(min mg) corresponding to the oxidation of about 600 NADH per second. The rate was virtually identical when the reaction was started either by an addition of NADH (Fig. 2a) or the enzyme (data not shown). By contrast, when the reaction was started by ferricyanide addition, the initial rate dropped to only about 2.5% of the reaction rate obtained with the oxidised enzyme (Fig. 2a, the actual rates are provided in Supplementary Table 1). Again, the reaction rate accelerated over time in a turnover-dependent manner and reached a maximum rate just before the substrate was depleted, a finding that was unnoticed in previous studies^21, 22^. Rather it confirms the data obtained with *E. coli* cytoplasmic membranes (Fig. 1b). At a first glimpse, our data appear to be incompatible with the proposal of Sazanov and co-workers^22^ since the reaction as outlined therein should follow first-order and not second-order kinetics. Notably, the acceleration of the reaction rate depended on the substrate (data not shown) and protein concentrations. In comparison with the NADH initiated reaction, the reaction rate using 10 nm complex I peaked at 30% at non-limiting substrate concentrations. The diluted enzyme (2 nm complex I) reached only 20% of the NADH-initiated reaction (Fig. 2a). The rate acceleration can be explained by assuming that ferricyanide oxidises complex I cofactor(s) slightly faster than they are reduced by NADH. However, even at highest excess of ferricyanide over complex I, a reaction rate equivalent to the NADH-initiated reaction could not be obtained, indicating that under the assay conditions ferricyanide is not capable to fully oxidise complex I in the presence of NADH.

In the course of varying the enzyme concentration, it turned out that, at saturating substrate concentrations, the parabolic curve approximating a parallel to the y-axis, obtained with 5 nm complex I in the assay, became linear at 0.9 nm complex I and hyperbolic approximating the x-axis at lower protein concentrations (Fig. 2b). Thus, the reaction accelerated at complex I concentrations above 0.9 nm, while it faded out at lower concentrations. While the rate acceleration confirms the findings outlined above, the deactivation at sub-nanomolar concentrations might be caused by a decay of the highly diluted protein over the long reaction time, and concomitantly, a loss of FMN, as previously observed^22^. At around 1 nm of complex I, both effects appear to compensate each other, leading to a straight line (Fig. 2b). Accordingly, the reduced enzyme may still be prone to degradation even at higher concentrations, hence the proportion of irreversibly lost enzyme prior to re-oxidation remains elusive; but it likely accounts at least partly for the effect that re-oxidised complex I never achieved the fast reaction rates observed with the NADH-initiated enzyme. Coincidentally, Sazanov and co-workers conducted their experiments at enzyme concentrations of 1–2 nm ^22^, which is, according to our results, precisely at the boundary, where complex disintegration and oxidation-mediated regain of function are at balance. Consequently, such a setup will inevitably produce pseudo-first-order kinetics and it will miss the effect of cofactor oxidation on complex I activity upon reaching turnover conditions. Ultimately, the observed loss of activity cannot be simply attributed to a dissociation of FMNH_2_.

### Partial recovery of the activity by addition of exogenous FMN

**Figure 3:**
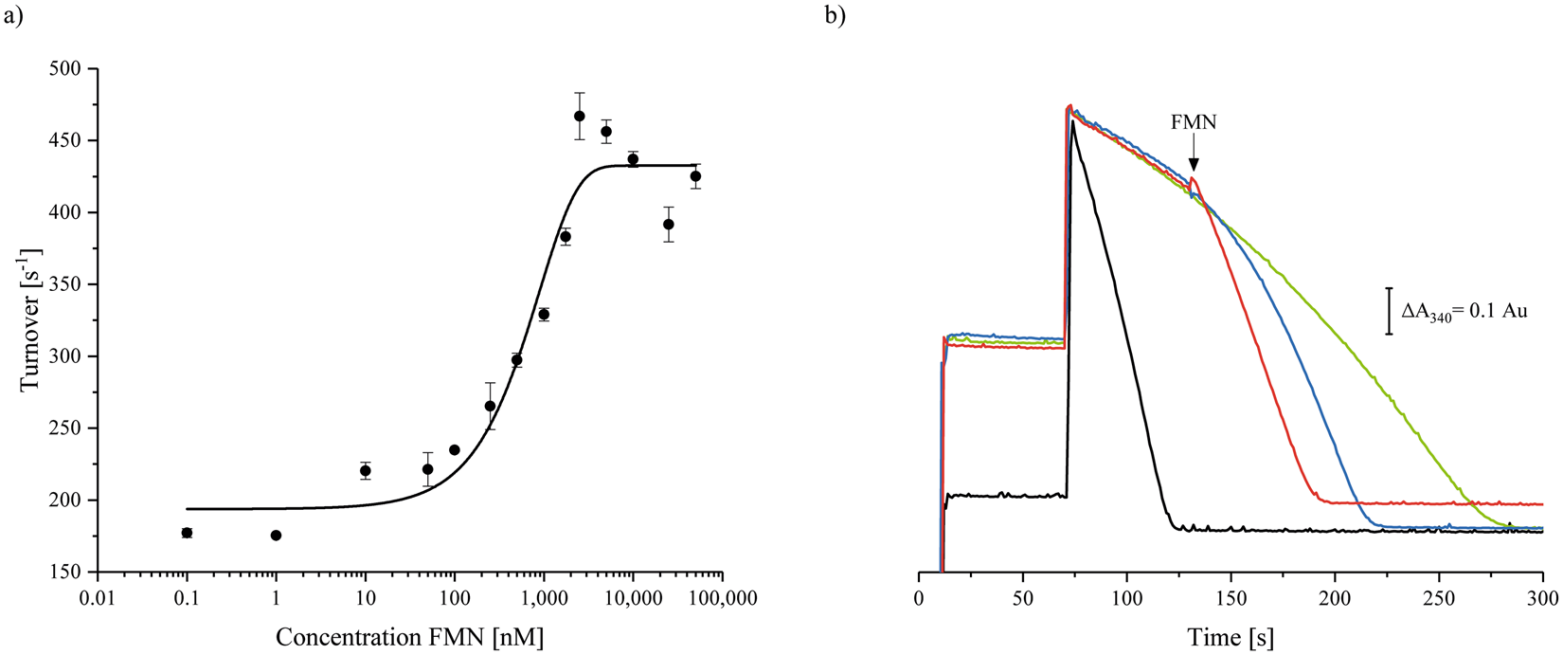
Activation of the NADH:ferricyanide oxidoreductase activity of complex I by externally added FMN. The assay contained 150 µm NADH and 10 nm *E. coli* complex I in 50 mm MES/NaOH, 50 mm NaCl, pH 6.0. The reaction was started by an addition of 1 mm ferricyanide and FMN was added after 60 sec. (**a**) The maximum activity obtained is plotted against the concentration of externally added FMN and the data points are fitted as a sigmoidal curve. (**b**) Shows the activity without an addition of FMN (green) and with an addition of either 1 µm (blue) or 10 µm (red) FMN (indicated by an arrow). The black curve shows the reaction started by an addition of NADH.

It was further reported that an addition of 2.5 µm exogenously added FMN to 1 nm complex reduced by 45 nm NADH recovered 60% of the NADH-initiated activity when the reaction was initiated with 1 mm ferricyanide^22^. In the light of our findings outlined above, we aimed at avoiding a concentration mediated enzyme decay and concomitant loss of FMN, and hence reduced 10 nm of complex I with 150 µm NADH, which is well above its *K*_m, app._ of 13 µm ^26^, and the reaction was started by an addition of 1 mm ferricyanide (Fig. 3). After 60 sec, 10 µm FMN were added to the assay leading to a prompt recovery of 73% of the activity of the NADH-initiated reaction (Fig. 3b). However, using lower FMN concentrations, such as 1 µm, a slow reaction was observed initially that gradually accelerated over time (Fig. 3b), as observed for the NADH:ferricyanide oxidoreductase activity (Fig. 2a). The initial rate of 60% of the maximum rate was only achieved after 20 sec (Fig. 3b). When titrating the activity with exogenous FMN, we obtained a sigmoidal curve that resembles the reported data^22^, however, half-maximal activation was observed not until 900 nm FMN were added (Fig. 3a). The difference between the values of half-maximal activation might be due to a degradation of the enzyme at the low protein concentration in the assay. The interpretation of the half-maximal activation as *K*_D_-value is not in agreement with the fact that a value 0.4 nm was reported for mitochondrial complex I at pH 9^27^. From the acceleration of the reaction after adding exogenous FMN we derive that it acts catalytically as redox mediator promoting the fast oxidation of reduced complex I cofactor(s) rather than associating with a supposedly empty FMN binding site.

### The low activity of the reduced enzyme is due to enhanced binding of NAD^+^

**Figure 4:**
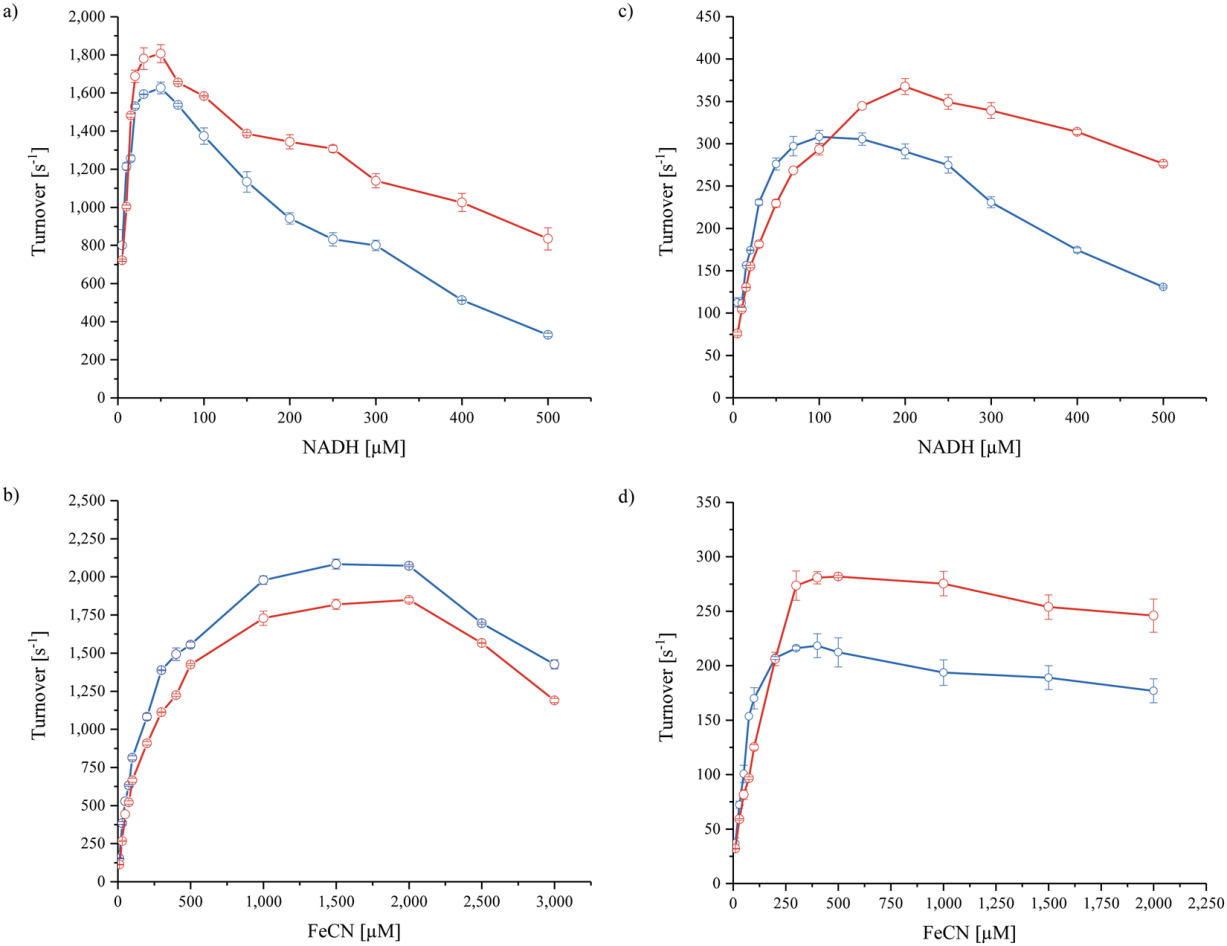
Dependence of the rate of *E. coli* complex I NADH:ferricyanide oxidoreductase activity on the NADH (**a**,**c**) and the ferricyanide (**b**,**d**) concentration. The reaction is either started by enzyme addition (**a**,**b**) or ferricyanide addition (**c**,**d**). The concentration of the enzyme was varied to give an appropriate rate for each reaction. The data in (**a**,**c**) were measured at 0.5 mm (blue) and 1 mm (red) ferricyanide and those in (**b**,**d**) at 50 µm (blue) and 100 µm (red) NADH. The steepness of the fast reaction just prior to substrate depletion was used to draw the plots (**c**,**d**).

Since our experiments suggested that the low velocity and the turnover-dependent acceleration of the NADH:ferricyanide reaction is governed by turnover-dependent cofactor re-oxidation, we next addressed the kinetic characterization of the NADH:ferricyanide oxidoreductase activity of *E. coli* complex I. It has been established that NADH competitively inhibits the reaction of the reduced enzyme with ferricyanide, while ferricyanide acts as competitor for NADH reacting with the oxidised enzyme^14^. The kinetic parameters *K*_m_, _app._^NADH^, *K*_i_, _app._^NADH^, and the rate constant for dissociation of bound NAD^+^ from the reduced enzyme were estimated in a previously described approach assuming a ping-pong-pong mechanism and ignoring the double substrate inhibition^14^. First, the reaction was started by an addition of the enzyme. The activity was inhibited at high concentrations of NADH and ferricyanide (Fig. 4a and b), confirming previous reports^13, 14, 16^. The apparent kinetic parameters were extracted from a double-reciprocal Lineweaver-Burk plot of the activity against the ferricyanide concentration (Supplementary Fig. S2) as described^14^. The intercepts of the Lineweaver-Burk fitting curves on the ordinate were plotted against the reciprocal of the NADH concentration enabling the calculation of *K*_m, app._^NADH^ and the rate constant for the dissociation of NAD^+^ from the reduced enzyme^14^. The slopes of the fitting curves plotted against the NADH concentration gave *K*_i, app._^NADH^. Starting the reaction with the enzyme, a *K*_m, app._^NADH^ of 11 µm and a *K*_i, app._^NADH^ of 22 µm were determined in good agreement with values obtained from the NADH:decyl-ubiquinone oxidoreductase activity of *E. coli* complex I^26, 28^. The *K*_m_^NADH^ for bovine heart complex I was determined to >200 µm ^13^. The discrepancy between the two values might relate to differences in the NADH concentration of roughly 300 µm in mitochondria but only about 80 µm in the *E. coli* cytoplasm^29, 30^. As expected from the proposed reaction scheme^14^ and a detailed kinetic analysis^10^, the rate of the NADH:ferricyanide oxidoreductase activity is mainly limited by hydride transfer and NAD^+^ dissociation. Here, the NAD^+^ dissociation constant was determined to 2.3 10^3^ s^−1^ (Table 1).

**Table 1:** Kinetic parameters of the NADH:ferricyanide oxidoreductase activity catalysed by the oxidised and the reduced *E. coli* complex I and the V96P/N142M^E^ variant, respectively, derived from the data shown in Figs. S2 and S3.

Reaction start	K_m, app._^NADH^ (µM)	K_i, app._^NADH^ (µM)	Rate constant: NAD^+^ dissociation (s^−1^)
Complex I	11	22	2.3 10^3^
FeCN	51	161	3.2 10^2^
V96P/N142M^E^	11	22	2.4 10^3^
FeCN	41	320	7.4 10^2^

The same set of experiments was performed with the reaction started by an addition of ferricyanide (Fig. 4c and d). Here, we also applied 10 nm complex I in the assay to minimise protein degradation and subsequent FMN release. As the reaction rate accelerated in a turnover-dependent manner during the individual measurements, the steepest slope just before substrate depletion is shown in Fig. 4c and d. Again, the activity was inhibited by high NADH and ferricyanide concentrations indicating the double substrate inhibition of the ping-pong-pong type reaction. When started with ferricyanide, higher NADH concentrations were needed to obtain maximum activity and to inhibit the reaction as compared to the reaction started by enzyme addition. By contrast, at a constant NADH concentration maximum activity was already observed at ferricyanide concentrations that were lower than in the initial assay started by addition of the enzyme (Fig. 4c and d). The initial slow reaction just after ferricyanide addition was used to calculate the kinetic parameters (Supplementary Fig. S2). However, as the state of the reaction is not well defined under these experimental conditions, the data were only used for a rough estimation. The *K*_m, app._^NADH^ was estimated to approximately 51 µm reflecting the low affinity of NADH to the reduced enzyme. This is in line with the reported *K*_Red_^NADH^, the affinity of NADH towards the reduced mitochondrial complex^13^. The *K*_i, app._^NADH^ was estimated to 161 µm, which expectedly is in the same range as the *K*_m, app._^NADH^ to the reduced enzyme. Importantly, the rate constant of the dissociation of bound NAD^+^ from the reduced complex was determined to 3.2 10^2^ s^−1^, which is more than sevenfold lower as compared to the reaction started by adding the enzyme (Table 1). This finding fits well to the initially low and subsequently accelerating reaction rate, when the following considerations are taken into account.

Complex I cofactors are reduced by NADH resulting in a stronger binding of NAD^+^. A following addition of ferricyanide leads to their partial re-oxidation, thus alleviating tight binding of NAD^+^. Since N1a has the most negative redox potential of all cofactors, it is first one to become re-oxidised. However, electrons are rapidly distributed between the FMN and Fe/S clusters^11^, keeping N1a partially reduced. Thus, the cofactors are not fully re-oxidised by ferricyanide due to the enhanced binding of NAD^+^ blocking ferricyanide binding. An uninhibited ferricyanide action, by contrast, would produce, after a limited acceleration phase, equal reaction rates for both ferricyanide and enzyme-initiated reactions, as observed in the experiment with the addition of exogenous FMN (Fig. 3a).

### Diminished NAD^+^ release is due to reduction of Fe/S cluster N1a

**Figure 5:**
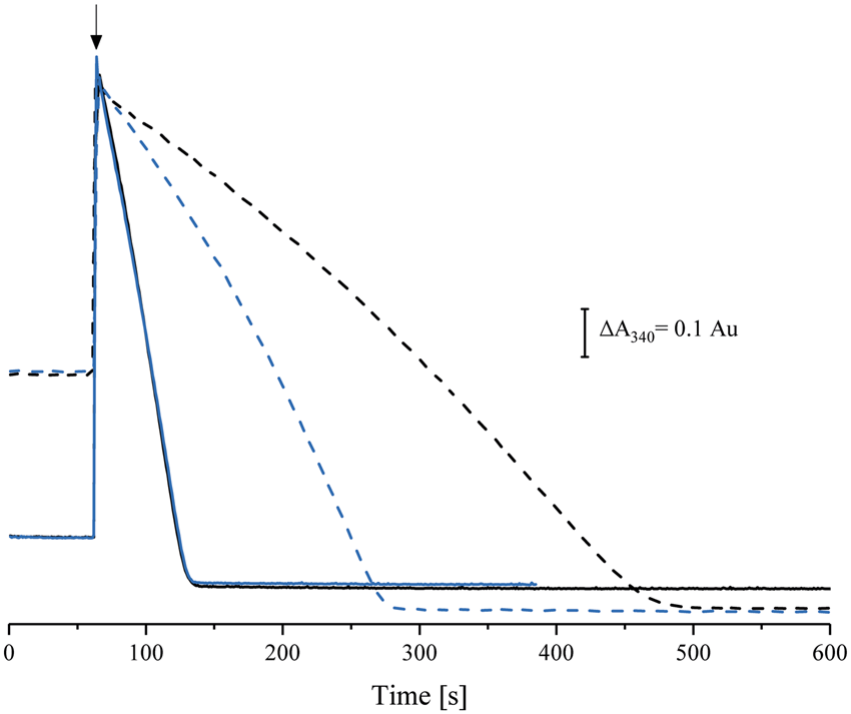
NADH:ferricyanide oxidoreductase activity of 10 nm isolated complex I (black) and 10 nm isolated V96P/N142M^E^ variant (blue). The assays contained 150 µm NADH and 1 mm ferricyanide in 50 mm MES/NaOH, 50 mm NaCl, pH 6.0. The reaction was either started by an addition of NADH (solid lines) or ferricyanide (dashed lines) when indicated (arrow).

Recently, an *E. coli* complex I variant was produced that featured a cluster N1a with a shifted midpoint potential of about −390 mV^12^. Two amino acids (V96P/N142M^E^, the superscript denotes the complex I subunit carrying the mutations) in the vicinity of the cluster were exchanged to residues that are found at homologous positions of the complex containing a ‘low-potential’ cluster N1a^12^. The EPR-spectroscopic properties of N1a in the V96P/N142M^E^ variant were insignificantly shifted and the rate of the NADH:ferricyanide oxidoreductase activity started by an addition of NADH was slightly diminished to 80% of the original activity indicating minor local changes in the environment of the cluster. Moreover, the NADH oxidase activity of the mutant membranes was also 80% of that of the original strain proving that the electron transfer from flavin to quinone was not significantly affected. Intriguingly, however, only 10% of the N1a population was reduced by 5 mm NADH. Under this experimental condition, the redox potential is set to approximately −370 mV and all other EPR-visible Fe/S clusters were fully detectable^12^. If now, as postulated above, N1a reduction caused enhanced binding of NAD^+^, this effect should be much less pronounced in the V96P/N142M^E^ variant. We tested this hypothesis by applying our established experimental setup to this variant and found that indeed, the NADH:ferricyanide oxidoreductase activity of the variant, when initiated with ferricyanide, achieved the maximum rate already after 90 sec, while it took twice as long for the wild type protein (190 sec, Fig. 5). In addition, the maximum activity of the variant doubled the one of the wild type protein. Finally, the maximum rate of the variant in the ferricyanide initiated reaction amounted to 45% of the NADH initiated reaction, while the wild type protein reached only 28% (Fig. 5). Consistently, the NAD^+^ dissociation constant was determined to 7.4 10^2^ (Table 1; Supplementary Fig. S3) which is more than twice than obtained with the original protein. These findings suggest that the oxidised state of the variant N1a shifts the equilibrium of the conformation of the NADH binding pocket from retaining NAD^+^ to displacing it.

### NAD^+^ binding to the catalytic site reduces the rate of ROS production

**Figure 6:**
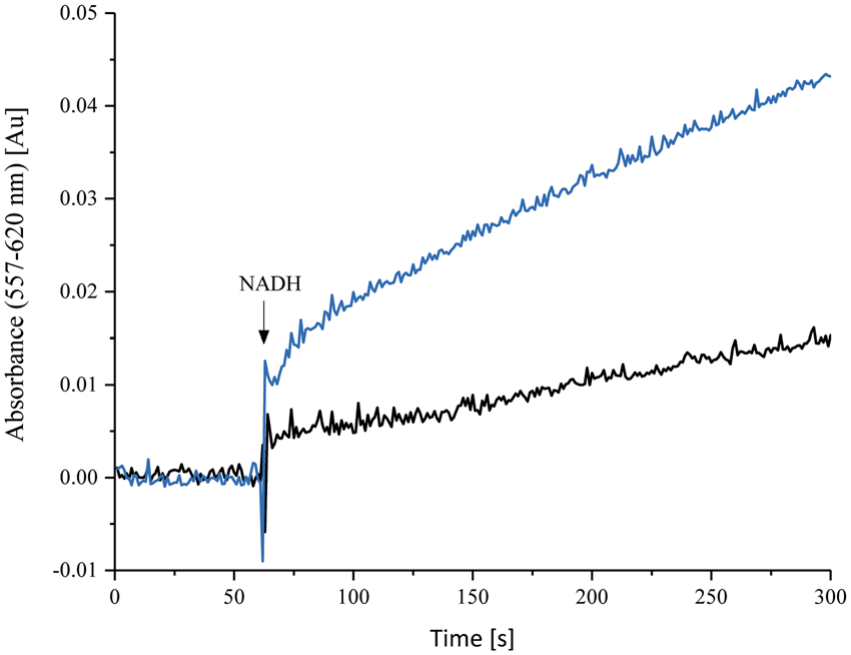
ROS-production by complex I (black) and the V96P/N142M^E^ variant (blue). H_2_O_2_ production was measured with the Amplex-red assay using 2 nm protein, horseradish peroxidase (2 U ml^−1^), 10 µm Amplex-red and 30 µm NADH.

It is well-known that ROS are produced by reduced FMN in complex I^31, 32^. Therefore, we addressed the question, on whether the enhanced binding of NAD^+^ caused by the reduction of N1a may prevent further reduction of the complex by NADH, ultimately precluding the production of ROS. Accordingly, we analyzed the ROS production of the V96P/N142M^E^ variant and compared it with the original enzyme. Our experiments showed a 1.6 fold higher ROS production by the variant than by the parental protein (Fig. 6). Thus, reduction of N1a in *E. coli* complex I contributes to the prevention of ROS production. The physiological relevance of this finding arises from the observation that N1a is reduced in *E. coli* complex I only when the most distal Fe/S cluster N2 is in the reduced state, *e.g*. when the quinone-pool is strongly reduced^11^. In this case N1a reduction would help to suppress ROS production at the FMN-site by promoting elsewise unfavoured NAD^+^ binding in order to occlude the entrance to the electron-rich active site for other electron acceptors, such as molecular oxygen. Upon oxidation of the quinone-pool, N1a would be oxidised, the NADH binding pocket would rapidly release NAD^+^, and thus become available for NADH. Finally, fast NADH oxidation activity would be restored.

## Discussion

In this study, we analysed the NADH:ferricyanide oxidoreductase activity of the respiratory complex I from *E. coli* in dependence of substrate and enzyme concentrations and differing orders of reagent additions. We found that the rate of the NADH:ferricyanide oxidoreductase activity of *E. coli* complex I in the NADH-reduced state is less than 5% of that in the oxidised state, as previously described^21, 22^. However, in contrast to a recent proposal^22^, we show that this effect cannot be easily assigned to a loss of FMNH_2_ from the complex. Instead, our experiments demonstrate that the rate of the reaction catalysed by the NADH-reduced enzyme accelerates in a turnover-dependent manner (Figs 1 and 2). Here, we find that exogenous FMN activates the reaction in a turnover-dependent manner with half-maximum activation at 900 nm FMN to 73% of the fast, NADH-initiated reaction (Fig. 3). Thus, it is likely that FMN acts as a redox mediator, extracting electrons from the reduced complex and delivering them to ferricyanide. This assumption is further supported by the fact that other redox mediators such menadione also accelerated the reaction although at slightly higher concentrations (Supplementary Fig. S4). The FMN-induced maximum NADH:ferricyanide oxidoreductase activity of 73% might indicate that about one quarter of the reduced preparation disintegrates (Fig. 3). This is in accordance with the finding that different types of the reaction curves are obtained at different protein concentrations in the assay (Fig. 2b). Using a diluted complex at sub-nanomolar concentrations, the reaction rate continuously slows down over time, indicating the enzymes’ decomposition and the subsequent release of FMN. Thus, loss of FMN is not causative for the slow reaction rate but a consequence of the degradation of the reduced and diluted enzyme.

An alternative hypothesis assigned the diminished activity to a conformational change at the NADH binding site^21^. In this early report a crude enriched fraction was used that most likely contained the NADH dehydrogenase fragment of *E. coli* complex^33^ in an approximately 10% purity. The authors concluded that the inactivation is due to a conformational change at the NADH oxidation but not the ferricyanide reduction site^21^. Today it is known that both substrates bind at the same site and that the reaction follows a ping-pong-pong mechanism^10, 13–20^. The data we present here are consistent with a NADH-induced local structural rearrangement of the protein at the NADH-binding site, influencing NAD^+^ release but not subsequent ferricyanide binding. This would prevent further binding of NADH by blocking the binding pocket with the reaction product NAD^+^. This assumption is supported by the sevenfold lower dissociation constant of NAD^+^ for the reduced enzyme as compared to the oxidised state (Table 1).

Our data indicate that the local structural rearrangement at the NADH binding site is most likely induced by the reduction of Fe/S cluster N1a: First, the ferricyanide activity is not diminished in *E. coli* cytoplasmic membranes when the Fe/S clusters are mostly in the oxidised state (Fig. 1A). The slow and accelerating activity of the complex is only detectable when the activity of the respiratory chain is inhibited by piericidin A or KCN (Fig. 1a). Accordingly, the addition of NADH leads to the reduction of N1a causing the low NADH:ferricyanide oxidoreductase activity. Second, the effect was specifically attributed to N1a by using the V96P/N142M^E^ variant in which N1a is hardly reduced by NADH^12^. Here, the throttling of the NADH:ferricyanide oxidoreductase activity upon NADH reduction was substantially diminished, although still detectable (Fig. 5). The dependence of the NADH:ferricyanide oxidoreductase activity on the order of the substrates added to the assay was not observed with complex I in *T. thermophilus* (Fig. 1)^22^ and in bovine heart mitochondria (Fig. 1). It is unlikely that differences in subunit composition between the bacterial and the mitochondrial complex are contributing to the slow reaction rate. Supportively, a comparison of the X-ray structure of the complex from *T. thermophilus* and bovine heart complex I determined by single-particle electron cyro-microscopy showed that in proximity of the NADH oxidation site there is no electron density arising from accessory subunits^34^.

Hence, the significant difference between the Fe/S clusters in complex I from *E. coli* on the one hand and *B. taurus* and *T. thermophilus* on the other hand arises from the redox potential of N1a^13, 35^. N1a is only reduced by NADH in complex I from *E. coli* ^23^, and in a flavoprotein subcomplex from *E. coli* ^23, 33^, *Aquifex aeolicus* ^36^ and *B. taurus* ^37^, however, it is not reduced by NADH in the fully assembled complex I from *B. taurus*, *Y. lipolytica*, *Pichia pastoris*, *Paracoccus denitrificans* and *T. thermophilus* ^38–41^. A simple explanation for these findings is the difference in the redox potential of the cluster between the species. In *E. coli* and *A. aeolicus* the redox potential of N1a was determined to −250 to −295 mV^12, 23, 36^. By contrast, in the complexes of all other species investigated this cluster has a much lower midpoint potential in the range of −370 to −420 mV, hence being not susceptive to reduction by NADH. However, it has to be taken into account that N1a was not reduced in the *B. taurus* holo-complex by an Eu(II) reagent with a very low potential of about −1 V^42^. This suggests that the cluster might be accessible neither for the solvent nor the flavin^41^, although N1a is located in electron tunnelling distance to the flavin^5–7, 43^.

We propose that the reduction of N1a leads to a conformational rearrangement within the NADH binding-site of *E. coli* complex I that induces an enhanced binding of NAD^+^ and simultaneously impedes further reduction of the complex by NADH. The physiological role could be the suppression of the production of ROS by complex I in its reduced state. Here, enhanced binding of NAD^+^ may either prevent the formation of FMNH_2_, the source of ROS in complex I^34, 35^ or it may impede binding of oxygen to the isoalloxazine ring. An inactivation of this proposed protective mechanism, as found in the V96P/N142M^E^ variant, leads to an enhanced ROS production (Fig. 6). Structural investigations of the complex in the reduced and the oxidised state need to be the subject of future studies to identify the proposed conformational rearrangement in the NADH binding pocket of *E. coli* complex I.

## Methods

### Expression strains and cell growth

For complex I production the *E. coli* strain BW25113Δ*nuo*Δ*ndh* with the *nuo*-operon coding all complex I genes and the *ndh* gene coding for the alternative NADH dehydrogenase being removed from the chromosome was used^44, 45^. BW25113Δ*nuo*Δ*ndh* was transformed with the complex I expression vector pBAD*nuo nuoF*_His_^45^ and grown aerobically in 1 L baffled flasks containing 400 mL autoinduction medium at 37 °C. Cells were harvested in the late exponential phase and stored at −80 °C. Cytoplasmic membranes were obtained as described^45^.

### Purification of complex I

Complex I was isolated from *E. coli* strain BW25113Δ*nuo*Δ*ndh*/pBAD*nuo nuoF*_His_ as described^45^. Briefly, membrane proteins were extracted using n-Dodecyl-ß-D-maltopyranoside (DDM, BioFroxx) and separated by anion exchange chromatography on Fractogel EMD TMAE Hicap (Merck). Fractions with NADH:ferricyanide oxidoreductase activity were pooled and further purified by affinity chromatography on Ni-IDA material (Invitrogen). Bound proteins were eluted in an imidazole gradient^45^. In addition to what has been described^45^, fractions from the affinity chromatography containing complex I were further purified on a 15 mL Source Q (GE Healthcare) column equilibrated in 50 mm MES/NaOH, 50 mm NaCl, 0.1% DDM, pH 6.0 at a flow rate of 1.5 mL/min. Bound proteins were eluted in a 40 mL linear gradient from 150–350 mm NaCl in 50 mm MES/NaOH, 0.1% DDM, pH 6.0. Peak fractions with NADH:ferricyanide oxidoreductase activity were combined and concentrated by ultrafiltration (Amicon Ultra-15, Millipore, 100 kDa MWCO) and stored in aliquots at −80 °C. Mutagenesis leading to the generation of the V96P/N142M^E^ variant is described^12^ and the variant protein was purified by the protocol described above.

### Preparation of bovine heart mitochondrial membranes

Mitochondria and mitochondrial membranes were isolated from bovine heart as described^46^. Mitochondrial membranes were suspended in 20 mm Tris/HCl, pH 7.4, 10% (v/v) glycerol, and 1 mm EDTA (final protein concentration: 12 mg mL^−1^) and directly used for enzymatic measurements at 30 °C.

### Preparation of cytoplasmic membranes from *T. thermophilus*

*T. thermophilus* cytoplasmic membranes were isolated as described^24^, suspended in 20 mm Tris/HCl, pH 7.4, 10% (v/v) glycerol, and 1 mm EDTA (final protein concentration: 12 mg mL^−1^) and directly used for enzymatic measurements at 60 °C.

### Determination of NADH:ferricyanide oxidoreductase activity

Kinetic measurements were performed by UV/vis-spectroscopy using a Tidas II Diode Array Spectrometer (J&M Analytik AG, Aalen). Assays were conducted at 30 °C or 60 °C in 1 mL 50 mm MES/NaOH, pH 6.0, 50 mm NaCl in a stirred cuvette^23^. NADH or d-NADH (both from Sigma-Aldrich) and ferricyanide (AppliChem) were added from stock solutions to the assay buffer and the reaction was started by an addition of either *E. coli* complex I or cytoplasmic and mitochondrial membranes, respectively. In a different series of experiments, the assay buffer either contained the protein and NADH and the reaction was started by an addition of ferricyanide or the assay buffer contained ferricyanide and the enzyme and the reaction was started by an addition of NADH. All enzymatic activities were corrected for the non-enzymatic reaction of NADH with ferricyanide that accounted to 2–4% of the enzymatic activity depending on the concentrations. Routinely, the assay contained 1 mm potassium ferricyanide and 150 µm NADH or d-NADH. To determine the kinetic parameters, the NADH:ferricyanide oxidoreductase activity in the presence of 0.25, 0.33, 0.5, 1.0 and 2.0 mm ferricyanide was titrated with 20, 50, 100, 150 and 200 µm NADH, respectively. The activity was observed at 340 nm following NADH oxidation (ɛ = 6.2 mM^−1^ cm^−1^). When indicated, 10 µm piericidin A (Sigma) or 20 mm KCN (Roth) were added to cytoplasmic or mitochondrial membranes.

### Amplex Red assay

The formation of ROS was determined by the Amplex Red assay as described^31^. The NADH concentration was 30 µm and the assay contained 5 µg isolated complex I in 1 mL (10 nm).

## Electronic supplementary material

Supplementary Information

## References

[1] Brandt U (2006). Energy converting NADH:quinone oxidoreductase (complex I). Annu. Rev. Biochem..

[2] Hirst J (2013). Mitochondrial complex I. Annu. Rev. Biochem..

[3] Sazanov, L. A. A structural perspective on respiratory complex I (ed. Sazanov, L. A.) (Springer, Dordrecht, 2012).

[4] Friedrich T (2014). On the mechanism of respiratory complex I. J. Bioenerg. Biomembr..

[5] Baradaran R, Berrisford JM, Minhas GS, Sazanov LA (2013). Crystal structure of the entire respiratory complex I. Nature.

[6] Zickermann V (2015). Mechanistic insight from the crystal structure of mitochondrial complex I. Science.

[7] Zhu J, Vinothkumar KR, Hirst J (2016). Structure of mammalian respiratory complex. Nature.

[8] Sharma V (2015). Redox-induced activation of the proton pump in respiratory complex I. Proc. Natl. Acad.Sci. USA.

[9] Ernster L (1965). Stereospecificity of certain soluble and particulate preparations of mitochondrial reduced nicotinamide-adenine dinucleotide dehydrogenase from beef heart. Nature.

[10] Birrell JA, Hirst J (2013). Investigation of NADH binding, Hydride Transfer, and NAD^+^ Dissociation during NADH Oxidation by Mitochondrial Complex I using Modified Nicotinamide Nucleotides. Biochemistry.

[11] De Vries S, Dörner K, Strampraad MFJ, Friedrich T (2015). Electron tunneling Rates in Complex I Are Tuned for Efficient Energy Conversion. Angew. Chem. Int. Ed..

[12] Birrell JA, Morina K, Bridges HR, Friedrich T, Hirst J (2013). Investigating the function of [2Fe-2S] cluster N1a, the off-pathway cluster in complex I, by manipulating its reduction potential. Biochem. J..

[13] Birrell JA, Yakovlev G, Hirst J (2009). Reactions of the Flavin Mononucleotide in Complex I: A Combined Mechanism Describes NADH Oxidation Coupled to the Reduction of ADAP^+^, Ferricyanide, or Molecular Oxygen. Biochemistry.

[14] Dooijewaard G, Slater EC (1976). Steady-state Kinetics of high-molecular weight (Type-I) NADH Dehydrogenase. Biochim. Biophys. Acta.

[15] Vinogradov AD (2008). NADH/NAD^+^ interaction with NADH:ubiquinone oxidoreductase (complex I). Biochim. Biophys Acta.

[16] Yakovlev G, Hirst J (2007). Transhydrogenase Reactions Catalyzed by Mitochondrial NADH-Ubiquinone Oxidoreductase (Complex I). Biochemistry.

[17] Zakharova NV, Zharova TV, Vinogradov AD (1999). Kinetics of transhydrogenase reaction catalyzed by the mitochondrial NADH-ubiquinone oxidoreductase (Complex I) imply more than one catalytic nucleotide-binding sites. FEBS Lett..

[18] Grivennikova VG, Kotlyar AB, Karliner JS, Cecchini G, Vinogradov AD (2007). Redox-dependent change of nucleotide affinity to the active site of the mammalian complex I. Biochemistry.

[19] Vinogradov AD, Grivennikova VG (2016). Oxidation of NADH and ROS production by respiratory complex I. Biochim. Biophys. Acta.

[20] Birrell JA, King MS, Hirst J (2011). A ternary mechanism for NADH oxidation by positively charged electron acceptors, catalyzed at the flavin site in respiratory complex I. FEBS Lett..

[21] Gutman M, Scheiter A, Avi-Dor Y (1968). The preparation and properties of the membranal DPNH dehydrogenase from *Escherichia coli*. Biochim. Biophys. Acta.

[22] Holt PJ, Efremov RG, Nakamaru-Ogiso E, Sazanov LA (2016). Reversible FMN dissociation from *Escherichia coli* respiratory complex I. Biochim. Biophys. Acta.

[23] Leif H, Sled VD, Ohnishi T, Weiss H, Friedrich T (1995). Isolation and characterization of the proton-translocating NADH: ubiquinone oxidoreductase from *Escherichia coli*. Eur. J. Biochem..

[24] Meinhardt SW (1990). Studies on the NADH-Menaquinone Oxidoreductase Segment of the Respiratory Chain in Thermus thermophilus MB-8. J. Biol. Chem..

[25] Bridges HR, Bill E, Hirst J (2012). Mössbauer spectroscopy on respiratory complex I: the iron-sulfur cluster ensemble in the NADH-reduced enzyme is partially oxidized. Biochemistry.

[26] Morina K (2011). Engineering the respiratory complex I to an energy-converting NADPH:ubiquinone oxidoreductase. J. Biol. Chem..

[27] Gostimskaya IS, Grivenikova VG, Cecchini G, Vinogradov AD (2007). Reversible dissociation of flavin mononucleotide from the mammalian membrane-bound NADH: ubiquinone oxidoreductase (complex I). FEBS Lett..

[28] Friedrich T (1994). Two binding sites of inhibitors in NADH: ubiquinone oxidoreductase (complex I). Eur. J. Biochem..

[29] Williamson JR, Corkey BE (1979). Assay of citric acid cycle intermediates and related compounds. Methods Enzymol..

[30] Bennett BD (2009). Absolute metabolite concentrations and implied enzyme active site occupancy in *Escherichia coli*. Nat. Chem. Biol..

[31] Kussmaul L, Hirst J (2006). The mechanism of superoxide production by NADH:ubiquinone oxidoreductase (complex I) from bovine heart mitochondria. Proc. Natl. Acad. Sci. USA.

[32] Dröse S, Brandt U (2012). Molecular mechanisms of superoxide production by the mitochondrial respiratory chain. Adv. Exp. Med. Biol..

[33] Braun M, Bungert S, Friedrich T (1998). Characterization of the overproduced NADH dehydrogenase fragment of the NADH:ubiquinone oxidoreductase (complex I) from *Escherichia coli*. Biochemistry.

[34] Vinothkumar KR, Zhu J, Hirst J (2014). Architecture of mammalian respiratory complex I. Nature.

[35] Gnandt E, Dörner K, Strampraad MF, de Vries S, Friedrich T (2016). The multitude of iron-sulfur clusters in respiratory complex I. Biochim. Biophys. Acta.

[36] Kohlstädt M (2008). Heterologous production, isolation, characterization and crystallization of a soluble fragment of the NADH:ubiquinone oxidoreductase (complex I) from *Aquifex aeolicus*. Biochemistry.

[37] Ragan CI, Galante YM, Hatefi Y, Ohnishi T (1982). Resolution of mitochondrial NADH dehydrogenase and isolation of two iron-sulfur proteins. Biochemistry.

[38] Zu Y, Di Bernardo S, Yagi T, Hirst J (2002). Redox properties of the [2Fe-2S] center in the 24 kDa (NQO2) subunit of NADH:ubiquinone oxidoreductase (complex I). Biochemistry.

[39] Maly T (2006). Cluster N1 of complex I from *Yarrowia lipolytica* studied by pulsed EPR spectroscopy. J. Biol. Inorg. Chem..

[40] Bridges HR, Grgic L, Harbour ME, Hirst J (2009). The respiratory complexes I from the mitochondria of two Pichia species. Biochem. J..

[41] Meinhardt SW, Kula T, Yagi T, Lillich T, Ohnishi T (1987). EPR characterization of the iron-sulfur clusters in the NADH: ubiquinone oxidoreductase segment of the respiratory chain in *Paracoccus denitrificans*. J. Biol. Chem..

[42] Reda T, Barker CD, Hirst J (2008). Reduction of the iron-sulfur clusters in mitochondrial NADH:ubiquinone oxidoreductase (complex I) by EuII-DTPA, a very low potential reductant. Biochemistry.

[43] Sazanov LA, Hinchliffe P (2006). Structure of the hydrophilic domain of respiratory complex I from *Thermus thermophilus*. Science.

[44] Datsenko KA, Wanner BL (2000). One-step inactivation of chromosomal genes in *Escherichia coli* K-12 using PCR products. Proc. Natl. Acad.Sci. USA.

[45] Pohl T, Uhlmann M, Kaufenstein M, Friedrich T (2007). Lambda Red-mediated mutagenesis and efficient large scale affinity purification of the *Escherichia coli* NADH:ubiquinone oxidoreductase (complex I). Biochemistry.

[46] Kotlyar AB, Vinogradov AD (1990). Slow active/inactive transition of the mitochondrial NADH-ubiquinone reductase. Biochim. Biophys. Acta.

