# Supplementary material for: Reduction of the off-pathway iron-sulphur cluster N1a of Escherichia coli respiratory complex I restrains NAD+ dissociation
Source: Sci Rep. 2017 Aug 18;7:8754. doi: 10.1038/s41598-017-09345-4 (PMC5562879; doi:10.1038/s41598-017-09345-4)
Supplement: Supplementary file 1 — Supplementary Information [file 41598_2017_9345_MOESM1_ESM.doc]

Supplementary Information for:

**Reduction of the off-pathway iron-sulphur cluster N1a of *Escherichia coli* respiratory complex I restrains NAD^+^ dissociation**

Emmanuel Gnandt, Johannes Schimpf, Caroline Harter, Jo Hoeser, Thorsten Friedrich^*^

Content:

Table S1: Actual NADH:ferricyanide oxidoreductase activities measured with complex I and the V96P/N142M^E^ variant when the reaction is either started by an NADH or ferricyanide addition.

Figure S1: Scheme of the cofactor localisation in complex I.

Figure S2: Determination of the kinetic parameters of the NADH:ferricyanide oxidoreductase activities obtained with complex I.

Figure S3: Determination of the kinetic parameters of the NADH:ferricyanide oxidoreductase activities obtained with V96P/N142M^E^ variant.

Figure S4: NADH:ferricyanide oxidoreductase activitiy of complex I in the presence of exogenous FMN and menadione.

Table S1: NADH:ferricyanide oxidoreductase activity of *E. coli* complex I and the V96P/N142ME variant in the presence of 150 µM NADH and 1 mM NADH. The activities were measured in 50 mm MES/NaOH, 50 mm NaCl, pH 6.0.

| Reaction start | Complex I | | V96P/N142M^E^ variant | |
| --- | --- | --- | --- | --- |
|  | µmol · mg^-1^ · s^-1^ | [%] | µmol · mg^-1^ · s^-1^ | [%] |
| NADH | 29.14 ± 0.45 | 100 | 27.97 ± 0.52 | 100 |
| FeCN (initial velocity) | 0.82 ± 0.01 | 2.8 | 6.3 ± 0.01 | 21.6 |
| FeCN (max. velocity) | 8.26 ± 0.21 | 28.0 | 12.56 ± 0.18 | 44.9 |


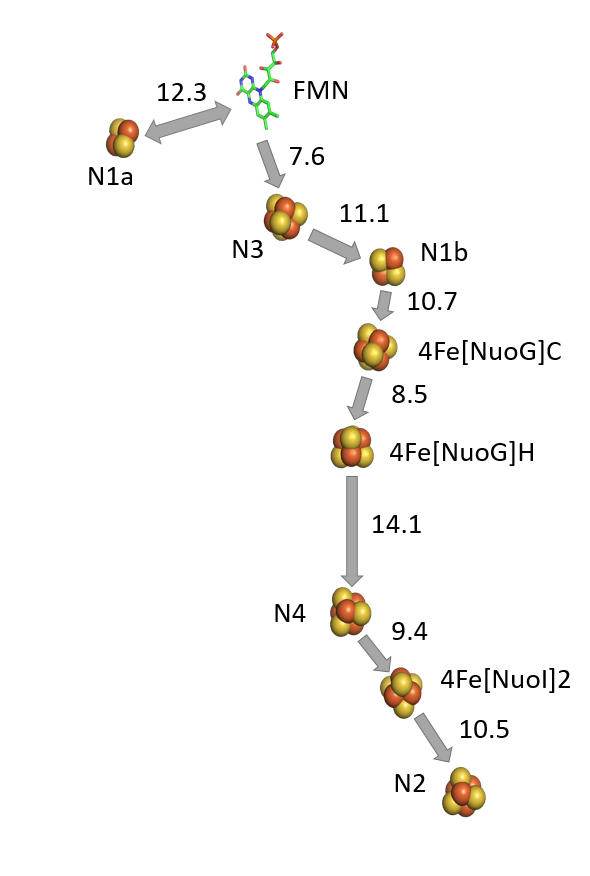


Figure S1: Scheme of the localisation of the Fe/S clusters in *E. coli* complex I based on the structure of the *T. thermophilus* complex^1^. The names of the individual Fe/S clusters involved in electron transfer and the distances between them in angstroms (from edge to edge) are provided^2^.


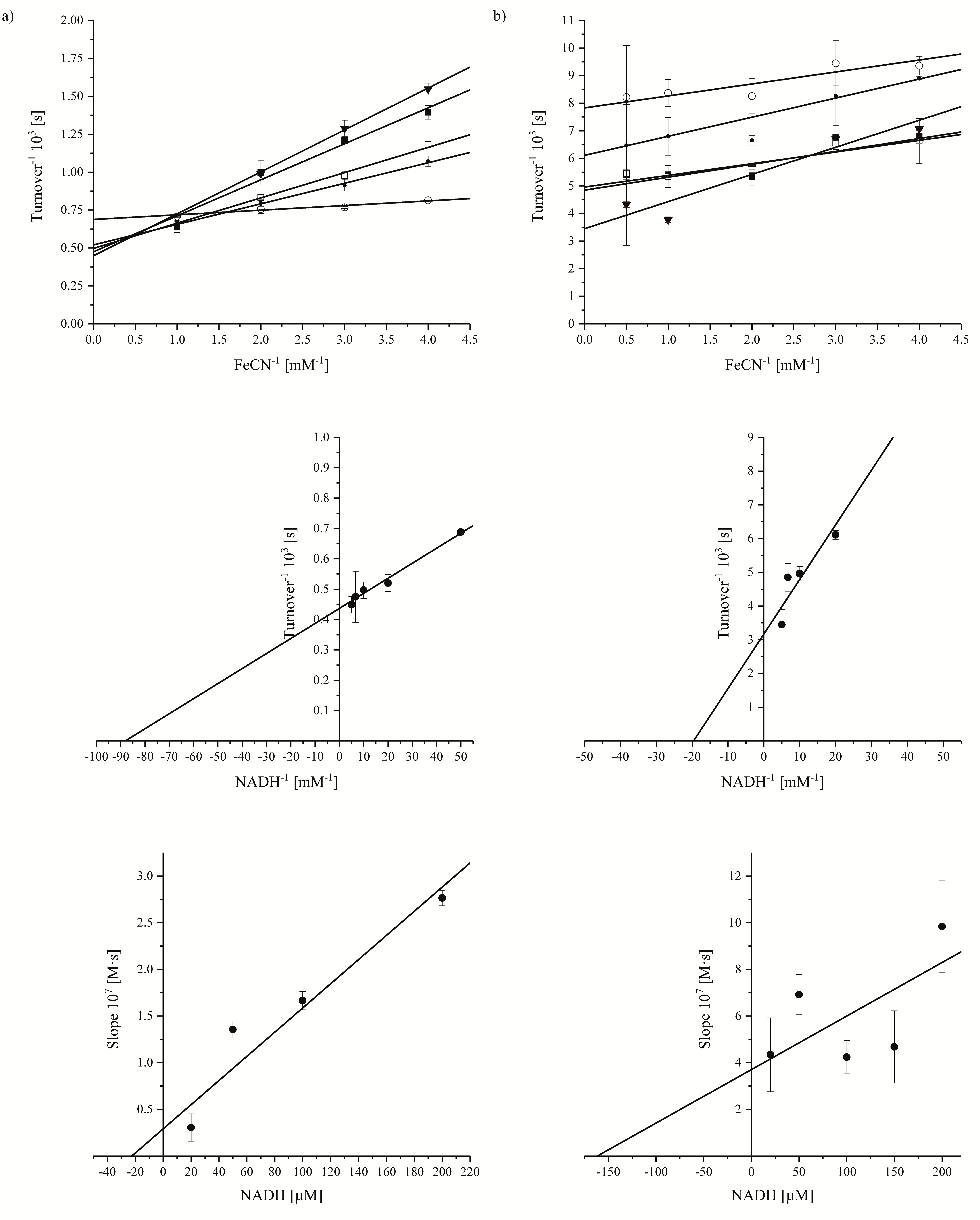


Figure S2: Lineweaver-Burk plot of the NADH:ferricyanide oxidoreductase activity of complex I when the reaction is started either by NADH (A) or ferricyanide (B) addition. The intercepts of the ordinate obtained in the Lineweaver-Burk plot are plotted against the reciprocal NADH concentration and the slopes against the NADH concentration to obtain the kinetic parameters. The concentration of the enzymes was varied to give an appropriate rate for each reaction. The steepness of the initial slow reaction just prior after substrate addition was used to draw the plots in (B). The activity was measured as function of the ferricyanide concentration at NADH concentrations of 20 (○), 50 (•), 100 (□), 150 (■) and 200 (▼) µm.


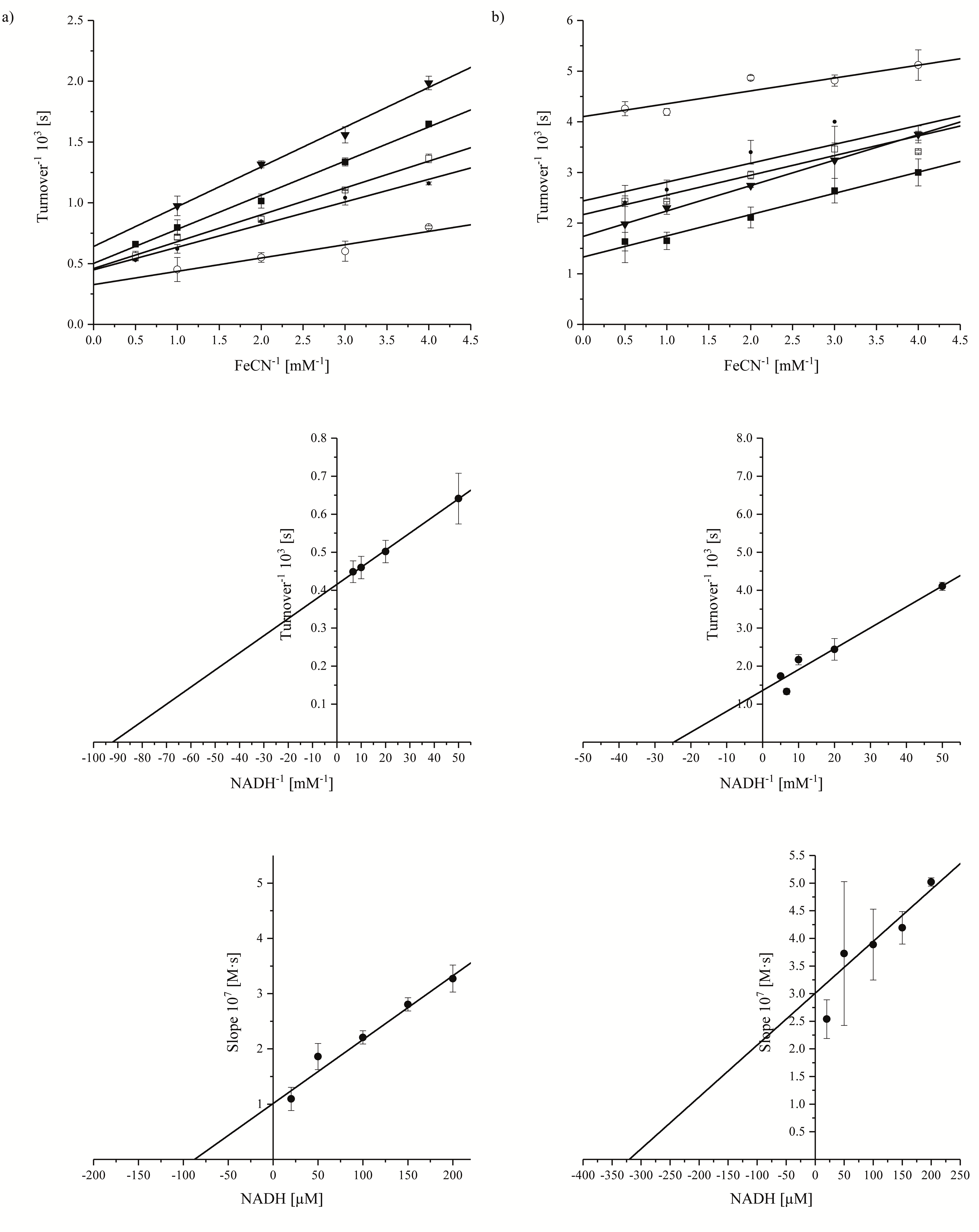


Figure S3: Lineweaver-Burk plot of the NADH:ferricyanide oxidoreductase activity of the V96P/N142M^E^ variant when the reaction is started either by NADH (A) or ferricyanide (B) addition. The intercepts of the ordinate obtained in the Lineweaver-Burk plot are plotted against the reciprocal NADH concentration and the slopes against the NADH concentration to obtain the kinetic parameters. The concentration of the enzymes was varied to give an appropriate rate for each reaction. The steepness of the initial slow reaction just prior after substrate addition was used to draw the plots in (B). The activity was measured as function of the ferricyanide concentration at NADH concentrations of 20 (○), 50 (•), 100 (□), 150 (■) and 200 (▼) µm.


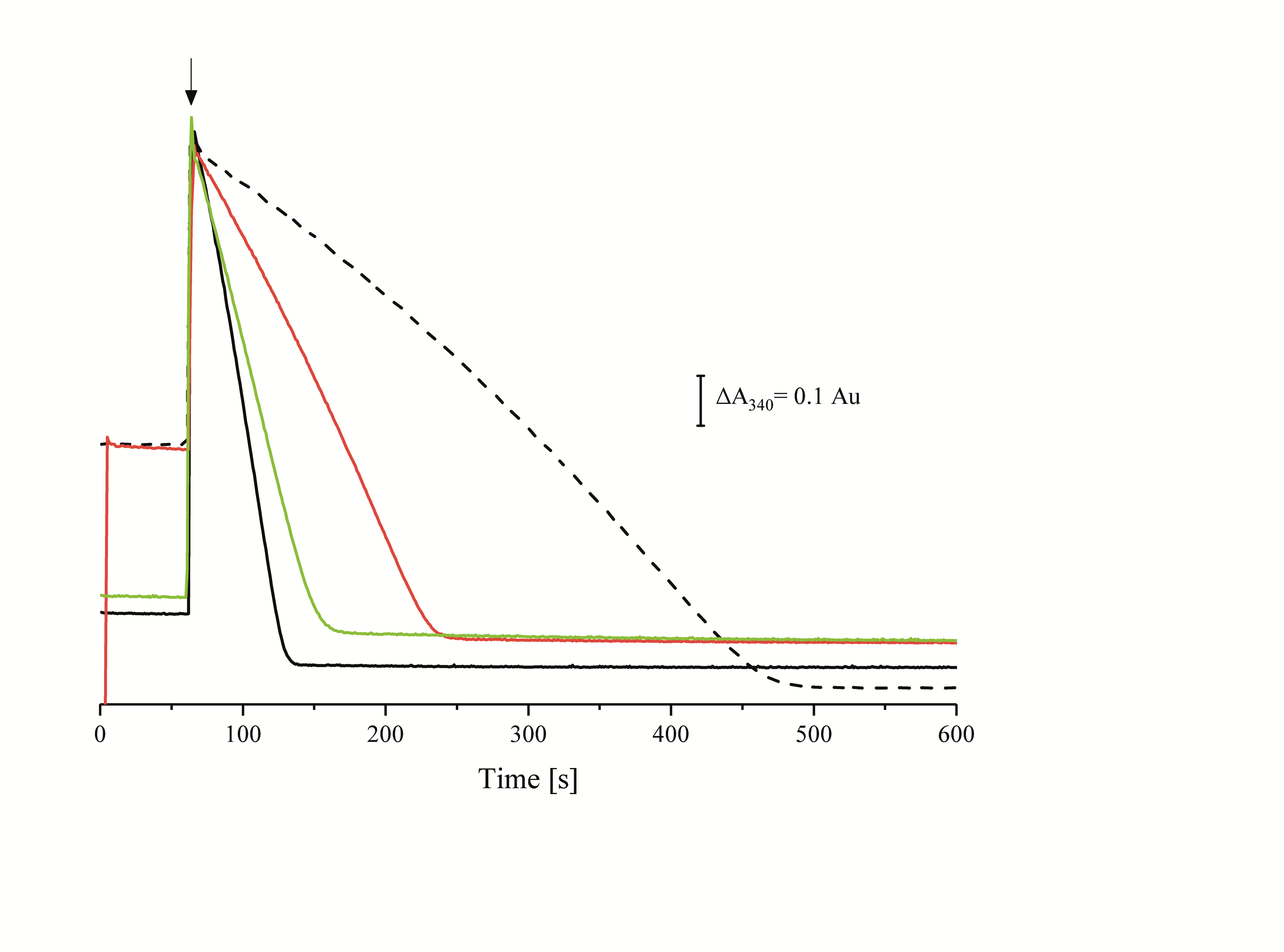


Figure S4: NADH/ferricyanide oxidoreductase activity of isolated *E. coli* complex I in the presence and absence of redox mediators. The activity of 10 nm isolated complex I initiated by NADH addition (black, solid line) and ferricyanide addition (black, dotted line) is shown. The assays contained 150 µm NADH and 1 mm ferricyanide in 50 mm MES/NaOH, 50 mm NaCl, pH 6.0. The activity of the reaction initiated by ferricyanide addition in the presence of 2.5 µm FMN and 2.5 µm menadione are shown in green and red, respectively.

References:

[1] Baradaran, R., Berrisford, J. M., Minhas, G. S. & Sazanov, L. A. Crystal structure of the entire respiratory complex I. *Nature* **494**, 441-445 (2013).

[2] Gnandt, E., Dörner, K., Strampraad, M. F., de Vries, S. & Friedrich, T. The multitude of iron-sulfur clusters in respiratory complex I. *Biochim. Biophys. Acta* **1857**, 1068-1072 (2016).
